# Effectiveness of Soft versus Rigid Back-Support Exoskeletons during a Lifting Task

**DOI:** 10.3390/ijerph18158062

**Published:** 2021-07-29

**Authors:** Mathilde Schwartz, Jean Theurel, Kévin Desbrosses

**Affiliations:** 1 Working Life Department, French National Research and Safety Institute for the Prevention of Occupational Accidents and Diseases (INRS), 54500 Vandœuvre-les-Nancy, France; mathilde.schwartz@inrs.fr (M.S.); jean.theurel@inrs.fr (J.T.); 2 Développement, Adaptation & Handicap (DevAH), University of Lorraine, 54000 Nancy, France

**Keywords:** musculoskeletal disorders, workload, wearable assistive device, occupational back-support exoskeleton, EMG, handling task, low back pain

## Abstract

This study investigated the influence of passive back-support exoskeletons (EXO_BK_) design, trunk sagittal inclination (TSI), and gender on the effectiveness of an exoskeleton to limit erector spinae muscle (ES) activation during a sagittal lifting/lowering task. Twenty-nine volunteers performed an experimental dynamic task with two exoskeletons (two different designs: soft (SUIT) and rigid (SKEL)), and without equipment (FREE). The ES activity was analyzed for eight parts of TSI, each corresponding to 25% of the range of motion (lifting: P1 to P4; lowering: P5 to P8). The impact of EXO_BK_ on ES activity depended on the interaction between exoskeleton design and TSI. With SKEL, ES muscle activity significantly increased for P8 (+36.8%) and tended to decrease for P3 (−7.2%, *p* = 0.06), compared to FREE. SUIT resulted in lower ES muscle activity for P2 (−9.6%), P3 (−8.7%, *p* = 0.06), and P7 (−11.1%), in comparison with FREE. Gender did not influence the effect of either back-support exoskeletons on ES muscle activity. These results point to the need for particular attention with regard to (1) exoskeleton design (rigid versus soft) and to (2) the range of trunk motion, when selecting an EXO_BK_. In practice, the choice of a passive back-support exoskeleton, between rigid and soft design, requires an evaluation of human-exoskeleton interaction in real task conditions. The characterization of trunk kinematics and ranges of motion appears essential to identify the benefits and the negative effects to take into account with each exoskeleton design.

## 1. Introduction

Occupational exoskeletons are wearable devices generally aimed at supporting users in performing their work tasks, by generating appropriate force/torque on one or multiple human joints. There has been increasing interest in employing exoskeletons for workplace ergonomics, particularly with the aim of reducing physical workload [1] and risk of musculoskeletal disorders (MSD) for workers [2]. Among these technologies, back-support exoskeletons (EXO_BK_) have been specifically designed in view of preventing the occurrence of low back pain (LBP). Much evidence has been reported in the scientific literature about the efficiency of passive EXO_BK_ in relation to limiting lumbar muscular stress during handling operations involving the trunk flexion/extension in the sagittal plane [1,2,3]. For examples, during laboratory studies, reductions in back muscle activity ranged from −10 to −44% for repetitive lifting [4] and from −10 [5,6] to −57% [7] in static postures. Research carried out in real work conditions has also revealed significant reductions (−20 to −25%) in spinal muscle activity when using a passive EXO_BK_ [8]. The recent systematic review on industrial back-support exoskeletons [3] evaluated a mean reduction in erector spinae muscles activity of −18% during lifting and −36% during static bending for passive EXO_BK_.

However, considerable disparities in the protocols of previous studies—including exoskeleton designs (e.g., flexible (soft) versus stiff (rigid) components), postures adopted (i.e., trunk inclination, and hip and knee flexion), loads lifted, task modalities (static versus dynamic), and populations—likely account for the substantial differences in the consequences on back muscle activity when using these systems [3]. As a result, these differences have created confusion regarding the benefits to be expected when using an EXO_BK,_ in terms of back muscle loading. Three sources of variability seem to be of particular importance.

First, the differences in the magnitude of reductions of muscular activity can vary depending on the passive EXO_BK_ designs [6,9]. More particularly, the assistive torque delivered by passive EXO_BK_ can be ensured either by elastic garments (exosuits, or soft exoskeletons) arranged in parallel with the spinal muscles, or by rigid spring elements (rigid exoskeletons) that act as hip extensions [10]. These components are tensioned when the trunk bends forward in the sagittal plane, and they then prompt the body to straighten up and adopt its initial position. However, to our knowledge, there is no information available on the influence of soft versus rigid exoskeleton designs on the efficiency of assistive devices in reducing spinal muscle activity during a similar trunk sagittal bending task [3]. Contrary to exosuits, the tensioning mechanisms of rigid EXO_BK_ are generally located at the hip level [6,9,11], thus partially neglecting independent hip and lumbar flexion in humans. In addition, the location of the contact point between the device and the user could also have impacts on posture and hence on the activity of muscles involved in posture regulation. For example, it appears that using a soft exoskeleton can significantly affect spine kinematics during lifting, by reducing lumbar and thoracic flexion [12]. Abdoli et al. [13] found a significant increase in plantar flexion during lifting tasks. Using a rigid EXO_BK_ (Laevo V1), Bosch, van Eck, Knitel, and de Looze [11] showed an increase in knee extension during forward bending.

Second, the benefits obtained from EXO_BK_ regarding back muscle activity appear to be dependent on the posture adopted (i.e., trunk sagittal inclination) during the experimental tasks performed. In the study by Lamers et al. [14], the reductions in back muscle activity observed with a passive custom-made EXO_BK_ varied from −23 to −43% for leaning tasks, performed at 30 and 90° respectively, in flexion (with a 4 kg load). In the study by Koopman, Kingma, Faber, de Looze, and van Dieen [7], the reductions in back muscle activity observed during static bending tasks with a passive EXO_BK_ (Laevo V2) varied from −11 to −57%, depending on the experimental postures imposed (five different hand heights). Moreover, it appeared that the relationships between the amplitude of muscle activity reduction and trunk posture were highly variable across subjects, probably as a function of individual kinematics and/or anthropometric characteristics [7,15].

Third, gender may also affect the relative benefits of using a passive exoskeleton. Indeed, some studies have shown different changes on lumbar muscles activity between men and women [6,9,16]. For example, So, Cheung, Liu, Tang, Tsoi, and Wu [16] noted a reduction in EMG activity of the erector spinae (ES) muscles with an EXO_BK_ for men but no change for women. Alemi, Madinei, Kim, Srinivasan, and Nussbaum [6] also reported an interaction between EXO_BK_ models and gender. In their study, the trunk extensors muscles activity was significantly decreased for women when using the Laevo V2.5 and BackX exoskeletons during a repetitive lifting/lowering task, while men noted a decrease only with the BackX.

Considering these sources of variability in back muscles activity, the objective of this study was to assess the influence of (1) the exoskeleton design (soft versus rigid exoskeleton), (2) the posture adopted (different trunk sagittal inclination), (3) the gender, and (4) the interactions between these parameters on the effectiveness of using a passive EXO_BK_ in terms of lumbar muscle activation reductions during a dynamic lifting/lowering task.

## 2. Materials and Methods

### 2.1. Participants

Twenty-nine volunteers (15 men: 23 ± 3 years, 179 ± 6 cm, 77 ± 7 kg; and 14 women: 22 ± 2 years, 167 ± 4 cm, 58 ± 9 kg) without back pathologies volunteered to participate in this study. All the participants followed a standardized training protocol including functional and handling tasks with and without an exoskeleton. The training protocol consisted of three 20 min sessions for each assistive condition (wearing soft and rigid exoskeletons and without wearing it). Participants gave their written consent after receiving detailed information on the objectives, protocol, and possible risks of the study. The experimental protocol received approval from the ethical committee (no. IDRCB 2017-1702538-45). Each volunteer participated in the present study following a medical examination.

### 2.2. Experimental Design

**Figure 1:**
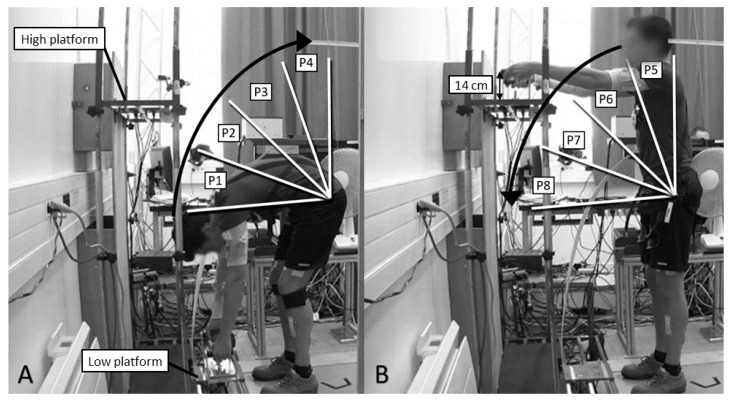
Experimental task performed. The range of motion of trunk sagittal inclination (TSI) was divided into four equal parts for the lifting action (**A**: low (P1) to high platform (P4)) and for the lowering action (**B**: high (P5) to low platform (P8)).

The participants had to perform a load lifting/lowering task (LLT) with a soft back-exosuit (SUIT), a rigid back-exoskeleton (SKEL), and without assistance (FREE). The LLT was a standardized task consisting in lifting a load (8 kg) from a low platform to a high one, and vice versa for 30 s at an imposed rate, using a rhythmic beep (20 beeps/minute, in order to perform 5 full cycles). One full cycle included both actions of lifting and lowering the load. Both platforms were facing the participant so as to limit the movement to the sagittal plane. These platforms were installed according to the anthropometric characteristics of the participants, at ankle height and shoulder height minus 14 cm (this value corresponded to the height of the load handles). The high platform was positioned behind the low one to obtain complete elbow extension in the sagittal plane (Figure 1). The platform’s position was strictly identical for the three experimental exoskeleton conditions (FREE, SKEL, and SUIT). The participants were instructed to hold their knee almost straight (i.e., from 5 to 10° flexion) but without locking the joint. Position of the feet was fixed, at shoulder width, for each participant by using tape on the floor to ensure a similar placement between conditions. The participants repeated the task twice for the three exoskeleton conditions (FREE, SKEL, and SUIT). The order of these exoskeleton conditions was randomized over subjects. A minimal recovery period of 30 s was imposed after each trial, and 5 min after each exoskeleton condition.

### 2.3. Exoskeletons

**Figure 2:**
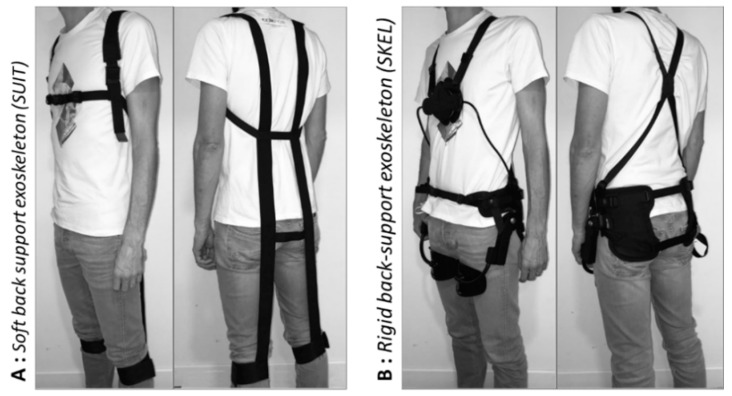
The two exoskeletons used; (**A**) soft back-support exoskeleton or exosuit (SUIT) (Corfor^®^-V2, Villemus, France), and (**B**) rigid back-support exoskeleton (SKEL) (Laevo^®^-V1, Deft, The Netherlands).

*Soft back-support exoskeleton or exosuit (SUIT):* a passive textile-designed assistive device (Corfor^®^-V2, Villemus, France) was used. This exosuit has been designed to assist the ES muscles during manual material handling and static bending posture, using elastic energy stored during trunk flexion and expended during trunk extension. Two elastic elements are attached to shoulder straps at the upper ends, and under the knees at the lower ends (Figure 2A).

*Rigid back-support exoskeleton (SKEL):* a passive back-support exoskeleton (Laevo^®^-V1, Deft, The Netherlands) was used. This exoskeleton consists of 2 types of pad: one chest pad and two upper leg pads. On both sides of the body, the pads are connected through a circular tube with spring like characteristics. This exoskeleton is intended to assist the ES muscles, using energy stored in springs during trunk flexion and expended during trunk extension (Figure 2B).

### 2.4. Data Acquisition and Analyses

#### 2.4.1. Trunk Sagittal Inclination (TSI)

TSI was recorded using one wireless magneto-inertial measurement unit (MIMU, firmware version 2.0.8, Xsens, Enschede, The Netherlands). The sensor was placed on the trunk on the flat portion of the sternum. Data were recorded at 50 Hz, filtered using a 5 Hz low pass filter, and synchronized with all the other recorded data. The range of motion was calculated for the lifting action, and divided into four equal TSI parts (=25% of range of motion) (low (P1) to high platform (P4)) (Figure 1A). In the same manner, the range of motion was calculated for the lowering action, and divided into four equal TSI parts (high (P5) to low platform (P8)) (Figure 1B). In terms of TSI, P1 (lifting) corresponded to P8 (lowering), P2 to P7, P3 to P6, and P4 to P5. Throughout the movement, TSI covered a range from approximately 5 to 95° (0° corresponding to the gravity axis).

#### 2.4.2. Electromyography (EMG)

The EMG of the erector spinae (ES) muscles was continuously recorded on both sides (Cometa, Wave Plus™, Bareggio, Italy). Two single-use surface electrodes (BlueSensor N-00-S, Ambu) were placed on the skin in accordance with SENIAM recommendations [17]. The inter-electrode distance was 20 mm. The skin was prepared to maintain impedance lower than 5 kΩ. EMG signals were recorded at 2000 Hz, amplified (×1000), and filtered with a 10–500 Hz bandpass. A 30 Hz high pass filter was applied to remove the heart rate artefacts [18].

Before the first experimental task, two isometric submaximal contractions of ES muscles were performed. The participants were lying on a table, where only the lower body (hip and leg) was supported. They had to maintain a horizontal static posture for 5 s with the trunk facing the ground. All contractions were separated by a 1 min recovery. The highest 500 ms RMS (Root Mean Square) value was used as the reference value (RMS_REF_).

During each experimental condition, RMS was calculated over successive periods of 40 ms sliding windows in 0.5 ms steps. An averaged RMS value was calculated for each TSI part (from P1 to P8). Then, RMS values were expressed in percentage of the RMS_REF_, averaged over both sides of the body. In order to avoid any disturbance of the movement linked to the start or to the end of the task, cycles 1 and 5 were not selected for data analysis.

### 2.5. Statistical Analysis

The results are presented as means ± standard deviations (SD). EMG data were log-transformed for statistical analysis in order to achieve a normal distribution and were back-transformed to original units for presentation in the text and figures. For TSI and ES muscle activity, a three-way repeated ANOVA was used to assess the effect of exoskeleton (FREE, SKEL and SUIT), TSI parts (P1 to P8), gender (men and women), and their interactions. Significant effects were analyzed using post-hoc Tukey HSD pairwise comparisons. A 5% significance level was adopted (*p* < 0.05). Commercial software was used for theses analyses (Statgraphics Centurion XVI).

## 3. Results

Statistical analyses revealed significant (*p* < 0.05) main effects: (1) of the exoskeleton on both ES muscle activity and TSI, (2) of the TSI parts on the ES muscles activity and naturally on TSI, and (3) of the gender on the ES muscle activity (Table 1). Statistical analyses also evidenced interaction effect between (1) exoskeletons and TSI parts for ES muscle activity and TSI, (2) exoskeletons and gender for TSI, and (3) gender and TSI parts for ES muscles activity.

### 3.1. Main Effect

#### 3.1.1. Exoskeleton Effect

**Figure 3:**
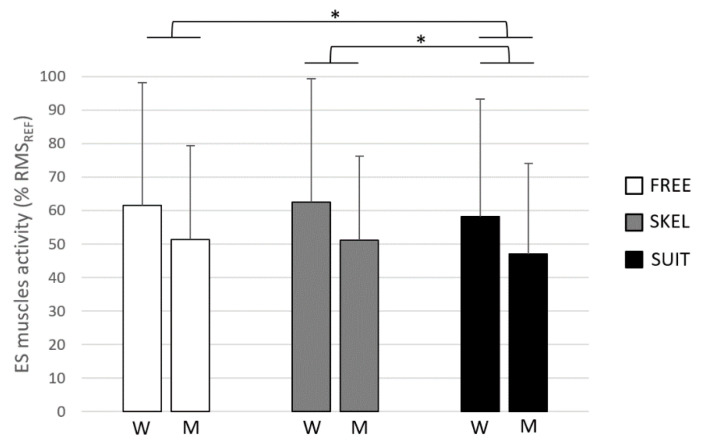
Averaged ES muscles activity (% RMS_REF_) and standard deviation in women (W) and men (M), with the use of the rigid exoskeleton (SKEL, gray), the exosuit (SUIT, black), and without equipment (FREE, white). *: significant differences between exoskeletons (*p* < 0.05). No interaction between exoskeleton and gender was reported.

Regarding overall movement (P1 to P8), the averaged ES muscle activity was significantly (*p* < 0.05) lower with the use of SUIT (52.5 ± 31.8% RMS_REF_) than without equipment (FREE: 56.5 ± 33.0% RMS_REF_) and SKEL (56.7 ± 31.9% RMS_REF_) (Figure 3). Concerning TSI, the averaged value was significantly greater for SKEL (50.9 ± 26.3°) than for FREE (49.0 ± 28.0°) and SUIT (49.1 ± 27.8°).

#### 3.1.2. Gender Effect

A significantly greater ES muscle activity was reported in women (60.7 ± 36.2% RMS_REF_) as compared to men (49.8 ± 26.8% RMS_REF_) on overall movement.

#### 3.1.3. TSI Part Effect

**Figure 4:**
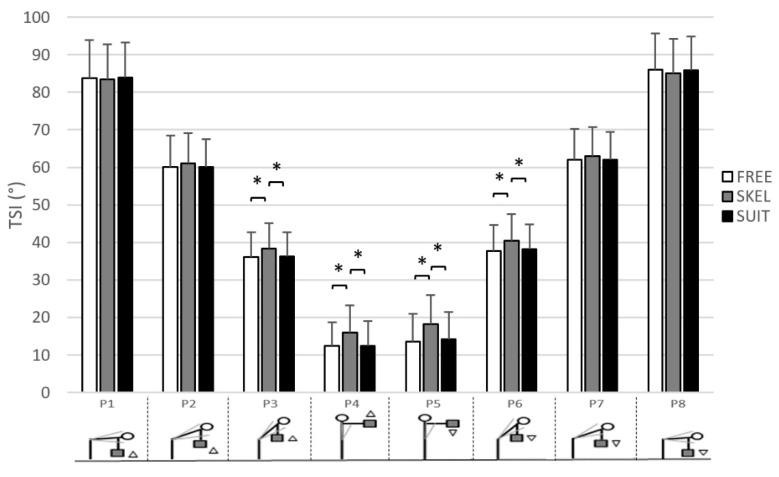
Averaged trunk sagittal inclination (TSI, in °) and standard deviation for each TSI part (from P1 to P8) of the lifting/lowering task (LLT) with the rigid exoskeleton (SKEL, gray), the exosuit (SUIT, black), and without equipment (FREE, white). 0° = straight posture. *: significant differences between exoskeletons (*p* < 0.05).

As required by our protocol, a main TSI part effect was obtained on TSI values (see Figure 4). Concerning averaged ES muscle activity, values were dependent on TSI parts: 34.3 ± 25.1% RMS_REF_ for P1, 63.8 ± 25.5 for P2, 83.6 ± 31.5 for P3, 88.1 ± 30.5 for P4, 57.8 ± 21.2 for P5, 44.9 ± 19.4 for P6, 45.8 ± 21.2 for P7, and 24.1 ± 17.3 for P8.

### 3.2. Interaction Effect

#### 3.2.1. Exoskeleton × TSI Part Interaction

With the use of SKEL, the averaged TSI was significantly greater than without equipment (FREE) and SUIT over half the range of motion (from P3 to P6) (Figure 4). There is not significant TSI difference between FREE and SUIT for all TSI parts.

**Figure 5:**
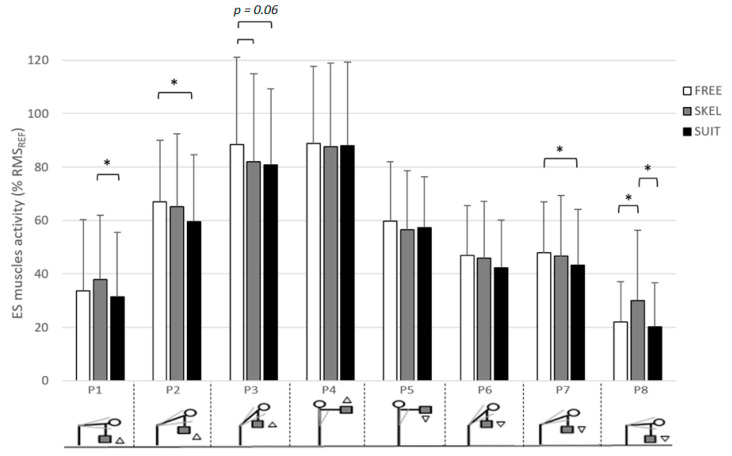
Averaged ES muscle activity (% RMS_REF_) and standard deviation for each TSI part (from P1 to P8) with the rigid exoskeleton (SKEL, gray), the exosuit (SUIT, black), and without equipment (FREE, white). *: significant differences between exoskeletons (*p* < 0.05).

Significantly higher ES muscle activity was observed with the use of SKEL (30.1 ± 19.0% RMS_REF_) during P8 compared to FREE (22.0 ± 16.9% RMS_REF_) (Figure 5). The use of SUIT resulted in significantly lower ES muscle activity compared to FREE for P2 (59.5 ± 25.1% RMS_REF_ versus 66.9 ± 23.1% RMS_REF_) and P7 (43.2 ± 21.0% RMS_REF_ vs. 47.8 ± 19.3% RMS_REF_) (Figure 5). During P3, the use of the two exoskeletons induced a slight but no significant (*p* = 0.06) lower ES muscle activity compared to FREE (SKEL: 82.0 ± 32.9% RMS_REF_, SUIT: 80.7 ± 28.6% RMS_REF_, FREE: 88.4 ± 32.7% RMS_REF_). For P1 and P8 TSI part, ES muscle activity was significantly lower for SUIT (P1: 31.5 ± 24.1% RMS_REF_ and P8: 20.2 ± 14.2% RMS_REF_) than for SKEL (P1: 37.8 ± 24.2% RMS_REF_ and P8: 30.1 ± 19.0% RMS_REF_).

#### 3.2.2. Exoskeleton × Gender

Without equipment (FREE), the averaged TSI on the overall movement was significantly greater in women (49.7 ± 28.8°) than in men (48.3 ± 27.2°). With SKEL, the averaged TSI was significantly lower in women (49.5 ± 27.0°) than in men (52.1 ± 25.6°). No significant difference was observed for the SUIT (49.0 ± 28.2° and 49.3 ± 27.4°, respectively, in women and men). For women, no significant difference was reported between the three-exoskeleton conditions, whereas for men, significant differences were observed between each condition.

#### 3.2.3. Gender × TSI Part

ES muscles activity was significantly greater in women than in men over most of the range of motion (from P3 to P8), with the exception of P1 and P2.

## 4. Discussion

The present study investigated the influence of exoskeleton design, trunk sagittal inclination (TSI), and gender on the impact of using wearable assistive devices on lumbar muscle activity during a dynamic forward lifting and lowering task (LLT). During LLT, the use of both EXO_BK_ resulted in significant changes in the averaged ES muscle activity compared to the control condition (FREE). These modifications depended not only on the exoskeleton design (SUIT versus SKEL), but also on the interaction between the exoskeleton design and parts of TSI. Gender did not modify these results.

### 4.1. Exoskeletons Design and TSI Effects

The use of SUIT resulted in a relative decrease of −7% of ES muscle activity during the overall LLT. These results appear to agree with those in the literature which also mostly reports significant reductions in back muscle activity during bending tasks with the use of a soft EXO_BK_ [2]. Nevertheless, the amplitude of the reductions of ES muscle activity here seems slightly lower in comparison with previous observations. For example, the reductions in back muscle activity when using exosuits during lifting tasks generally ranged from −10 to −40% [4,13,19,20,21,22]. However, most of the previous studies using exosuits did not specifically focus on the sagittal inclination of the trunk during the lifting task. When analyzing the present results for each TSI part, it appears that the decreases in ES muscle activity involved in the use of SUIT varied with phases of the movement (from −0.9 to −11.1%). More specifically, the relative reductions of ES muscle activity were not significant where the participants stood almost straight (P4 and P5, TSI ≈ 5–25°), and when they were strongly leaning (P1 and P8, TSI ≈ 75–95°). The beneficial effects of SUIT on ES muscle activity even appeared to be directly dependent on the parts of the TSI: approximately −8.7% for P3 (TSI ≈ 25–50°) (*p* = 0.06), and −11.1 and −9.6% for P2 and P7, respectively (TSI ≈ 50–75°). For the most extreme parts of the movement, the elastic garment may not be in its optimal operating range: not stretched enough on one side (P4 and P5) and close to its maximum stretch on the other side (P1 and P8), thereby reducing its effectiveness. Additionally, although not evaluated, a hysteresis could be present for P1 as demonstrated previously by Koopman, Kingma, Faber, de Looze, and van Dieen [7] for a rigid EXO_BK_, which can also reduce the support of SUIT. Finally, the contribution of ES muscle is reduced for large hip flexions. It is therefore likely that the effect of the system is relatively less visible in this position for this muscle. A more complete analysis of the back muscle chain could make it possible to assess whether other muscle activities were modified by SUIT at these trunk inclinations.

Contrary to most of the previous studies using exosuits, where a squat technique was advised for lifting tasks [14,21,22], the participants of the present study performed LLT with a stooped posture. Thus, it can be assumed that the tension (assistive force) in the elastic garment depends on the spine curvature and thus on the range of motion during stooped bending. Lamers, Yang, and Zelik [14] reported similar observations with the use of another soft EXO_BK_. The latter authors showed that the amplitude of back muscle activity reductions, compared to control conditions, increased with the TSI (−23% for a TSI close to 30°, −27% close to 60°, and −43% close to 90°). Comparing the amplitude of the decrease in ES muscle between the latter study [14] and the present one nevertheless remains difficult. Lamers, Yang, and Zelik [14] indeed recorded muscle activity only during static holding, and not during dynamic lifting. The modalities of both tasks can require the activation of different muscles. In addition, exoskeleton support can change between dynamic versus static action modalities [7].

Contrary to SUIT, the use of SKEL did not significantly change the result in ES muscle activity compared to the control condition, except for P8 where EMG increased. This result seems to run counter to the most common observations reported in the literature, on the impact of EXO_BK_ on ES muscle activity [2]. However, to our knowledge, only a few studies have specifically investigated the consequences of a rigid EXO_BK_ on back muscle activity during a dynamic bending task over a wide range of motions [6,7,9,23]. Nevertheless, other studies have also reported a lack of significant reductions of ES muscle activity when using rigid EXO_BK_ devices in stooped postures (TSI close to 70°) [5], and even slight increases of ES muscle activity when using the same exoskeleton (Laevo) in standing positions [7].

As the support delivered by such an exoskeleton is a function of angle [7], we expected that the effects of the SKEL during LLT would be related to the TSI. However, the benefits involved by SKEL in terms of back muscle activity reduction did not appear to be related to the increase of TSI. The slight and not significant (*p* = 0.06) reduction in ES muscle activity would seem to occur only during lifting, over P3 (−7.2%). The range of postures (≈25–50°) for which this exoskeleton involved this reduction in back muscle activity appears similar to that of previous studies that carried out experiments with the same exoskeleton. For example, Koopman, Kingma, Faber, de Looze, and van Dieen [7] recorded significant reductions of ES muscle activity (approximately −15%) during a stooped bending task with an exoskeleton at only 50% of the range of motion (0% corresponding to the floor, and 100% to standing upright). The latter results suggest that using such an exoskeleton involves reductions of ES muscle activity only over restricted ranges of trunk inclination, and not over a wide range of dynamic bending.

In terms of design, SKEL consists of two chest pads and two upper leg pads connected through a circular tube with spring-like characteristics. The flexion axis of the system is located at the level of the transversal axis of the hip. As a result, the support should be activated by hip flexion, and not directly by trunk inclination. However, in the present study, the participants adopted a stooped posture during LLT. Trunk inclination was not only associated with hip flexion, but also with spine flexion (i.e., curvature). Contrary to SUIT, SKEL cannot be directly tensioned by the spine curvature. Moreover, the use of SKEL may even tend to limit spine flexion [5,7]. In the present study, the lifting/lowering technique used by the subjects could thus partly explain the differences in the effectiveness of SKEL versus SUIT to reduce back muscle activation throughout the full range of motion.

Furthermore, counter intuitively, the use of SKEL resulted in a significant increase of ES muscle activity compared to FREE, when the TSI ranged from ≈75 to 95°, during the lowering phases (i.e., for P8). Several hypotheses can be made to explain this observation. First, this result could be linked to a change of spine kinematics and/or the coordination of spine and hip extensor muscles, when using SKEL during LLT, compared to FREE. Previous studies evidenced several changes in postural kinematics when using rigid EXO_BK_ [5], notably the Laevo [7,11], during dynamic lifting and static holding tasks. For example, Ulrey and Fathallah [5] reported a significant reduction of thoracic and lumbar flexion when using a similar rigid exoskeleton during static forward bending, in comparison with a control condition. Koopman, Kingma, Faber, de Looze, and van Dieen [7] also reported a significant reduction of hip flexion when using the Laevo exoskeleton during static holding tasks whereas the TSI remained similar both with and without an exoskeleton. In the present study, only TSI was recorded using an inertial unit located on the thoracic plexus. This hypothesis could not be verified. On the other hand, it can be assumed that the use of SKEL involved changes in hip and spine flexion, thereby affecting both the coordination and activity of the hip (e.g., gluteus maximus and biceps femoris) and spine (e.g., lumbar erector spinae and thoracic erector spinae) extensor muscles. In this case, the increase of ES muscle activity with SKEL could be due to an increase of the relative contribution of these muscles to spine erection. However further research is needed to confirm this hypothesis. Finally, considering the large hysteresis present in this device [7], we can assume that the support delivered by SKEL was particularly limited during the change of motion direction.

Ultimately, the interaction effect between the exoskeleton and TSI on ES muscle activity could be related to the technique used during the lifting/lowering task, and more particularly to the action needed to tension the passive EXO_BK_. It is probable that the effectiveness of SKEL to reduce back muscle efforts was related to hip flexion, while the effectiveness of SUIT was dependent on spine flexion. The present experimental protocol should be duplicated during a lifting task performed with a squat technique, involving a greater mobilization of the hip. The respective efficiency of both exoskeletons (soft versus rigid) would probably be different.

### 4.2. Gender Effects

In this study, we found a gender effect on the muscle activity of ES with a higher mean relative value for women. The weight of the load to be handled was the same for men and women (8 kg), and is likely to explain this difference, due to the lower average maximum voluntary force in women. Furthermore, no interaction between gender and EXO_BK_ was observed on back muscle activity. This result contrasts with previous studies showing differences in back extensor muscle activity between men and women when using an exoskeleton [6,9,16]. For example, Alemi, Madinei, Kim, Srinivasan, and Nussbaum [6] observed a reduction in muscle activity with the use of the two rigid EXO_BK_ tested (Laevo V2.5 and BackX) for women but only a reduction for one of the two EXO_BK_ (BackX) for men. Moreover, in this last study, these reductions were greater for women than for men. Another study [16], assessing a gender effect, showed a decrease in back extensor EMG activity with a rigid EXO_BK_ (BackX) only for men.

Firstly, we assume that these disparities in results between studies could be related to the task performed and the lifting technique used: lifting tasks with a free technique for Alemi, Madinei, Kim, Srinivasan, and Nussbaum [6], cardiopulmonary resuscitation chest compressions for So, Cheung, Liu, Tang, Tsoi, and Wu [16], and a LLT with a stoop technique for the present study. As gender can influence motor coordination during lifting tasks [24], particularly with greater hip flexion in women, it is possible that these protocols had a different impact on the biomechanical responses between men and women.

Secondly, it is probable that the design of exoskeletons could also contribute to this gender effect. Our results showed an interaction on trunk kinematics between gender and EXO_BK_. Women did not show any differences on the average TSI between the different exoskeleton conditions (FREE, SKEL, and SUIT), while men did. This gender effect may be due to variation in motor coordination [24] but also to different strategies for using exoskeletons. Men seemed to lean forward more with the exoskeletons, and this was more pronounced with the rigid one, as if they were seeking assistance through greater trunk support. The absence of this observation in women may be related to a greater perception of discomfort on their body when using exoskeletons. Although we did not evaluate this parameter, previous studies have reported differences in local discomfort between men and women when using EXO_BK_ [6,9,25]. Kozinc et al. [26] also pointed out that the discomfort threshold for pressure on the chest, thighs, or pelvis was lower in women. This parameter, related to the design of the exoskeleton, could therefore participate in the gender effect observed for the TSI.

In the present study, as there is no interaction between gender and EXO_BK_ on back muscles activity, the results on the effectiveness in terms of muscle activity reductions of the two EXO_BK_ tested appear to be valid for both men and women. However, to better understand these gender effects, further studies need to be conducted on the complete kinematics of the spine and hip during the use of different EXO_BK_.

### 4.3. Limitations

Several parameters of the experimental protocol had to be standardized in order to limit bias in the measurement of dependent variables. However, these same parameters can also be considered as limitations in the interpretation of the results. For example, both exoskeletons were examined during a dynamic lifting task. Since the dynamics of the torso (i.e., velocity/acceleration) could have an influence on ES muscle activity, this parameter was controlled by the frequency of the lift, which was identical for each experimental condition. As a result, the interpretation of the present results should be limited to this particular lifting speed. Nevertheless, this lifting speed was determined by the average of the free cadence chosen by the participants during the preliminary experiments. It can be assumed that this lifting speed was relatively realistic with regard to real handling tasks. In order to limit the impact of the interindividual heterogeneity in the present results, only young people participated in this study performed in the laboratory, using a highly controlled experimental task. Therefore, these conditions are not representative of the actual work context. The main objective was to test the interaction between exoskeleton design and TSI part on back muscle activity. It was thus necessary to perfectly control the experimental conditions and to study the exoskeletons while using them strictly for the purpose they have been designed. Finally, the use of normalized ranges of motion also appeared essential here to reduce interindividual differences and facilitate intercondition comparisons. Consequently, this methodological choice substantially reduced the practical applications of these results.

Furthermore, some of the methodological choices limited the scope of the analysis performed in this study. For example, only the ES muscle was examined during this study, while several other muscle groups contribute to the flexion/extension of the spine and the hip during stooped bending tasks. In practical terms, and for comparison with previous studies, the measure of ES is probably the most relevant choice. The ES muscle is systematically studied for back-support exoskeleton evaluations during laboratory and field research [3]. Moreover, the excessive effort of ES muscle is related to the occurrence of LBP. However, fundamentally, the study of the other muscles involved in the hip and spine mobilization would have provided useful information on changes in muscle coordination with the exoskeleton over the full range of motion. In addition, the use of only a single MIMU did not allow analyzing the coordination of pelvic and trunk movements. These measures would have allowed studying the influence of the exoskeleton design on the kinematics of the spine and pelvis. The lower limb kinematics were not studied either. Only knee flexion was controlled visually by the researcher during the experimentation, thus it cannot be excluded that slight changes occurred.

## 5. Conclusions

The present study showed that the impact of using an EXO_BK_ during a dynamic lifting/lowering task with an 8 kg load on ES muscle activity depended on both the exoskeleton design and TSI. The impact of the soft EXO_BK_ on the back muscle workload appeared beneficial between 25 to 75° of TSI during this task. On the contrary, the use of the rigid EXO_BK_ did not involve significant reductions of ES muscle activity during this dynamic task, but a significant increase when the TSI exceeded 75°. Gender did not affect these results on back muscle activity. It seems that the technique used during the lifting/lowering task could influence the performance of the EXO_BK_ in reducing back muscle activity. This technique could depend on the exoskeleton design, and more particularly on the action needed to tension it. It is probable that the effectiveness of the rigid exoskeleton to reduce back muscle efforts is related to hip flexion, while the effectiveness of the soft exoskeleton is dependent on spine flexion.

In practice, the choice of a passive back-support exoskeleton requires prior characterization of the tasks to which workers are exposed. Trunk kinematics and ranges of motion have to be considered in priority. Moreover, evaluations of human-exoskeleton interaction in task conditions remain essential to identify the benefits and potential negative effects of the exoskeleton on back muscle effort. These evaluations should also consider the influence of gender on the consequences induced by the use of exoskeletons.

## Figures and Tables

**Table 1:** Statistical fixed effects on TSI and ES muscle activity from the ANOVA.

		TSI		ES Muscles Activity	
**Main Effects**	**DoF**	**F**	***p***	**F**	***p***
Exoskeleton	2	18.88	**<0.001**	16.60	**<0.001**
Gender	1	2.71	0.1	121.24	**<0.001**
TSI part	7	6528.25	**<0.001**	519	**<0.001**
**Interaction effects**	**DoF**	**F**	***p***	**F**	***p***
Exoskeleton x TSI part	14	3.16	**<0.001**	5.39	**<0.001**
Exoskeleton x Gender	2	16.63	**<0.001**	1.96	0.14
Gender x TSI part	7	1.17	0.31	6.72	**<0.001**
Exoskeleton x Gender x TSI part	14	0.21	1	1.41	0.14

DoF: degrees of freedom. Significant effects (*p* < 0.05) are presented in bold type.

## Data Availability

The data presented in this study are available on request from the corresponding author. The data are not publicly available due to ethical regulations.
